# Orthopaedic Practices and Surgeries during COVID-19 in Pakistan - A Survey Based Study

**DOI:** 10.5704/MOJ.2103.011

**Published:** 2021-03

**Authors:** M Saad-Ilyas, U Zehra, UU Khan, I Mohammad, R Muhammad, A Aziz

**Affiliations:** 1 Department of Orthopaedics & Spine Surgery, Ghurki Trust Teaching Hospital, Lahore, Pakistan; 2 Department of Anatomy, University of Health Sciences, Lahore, Pakistan; 3 Department of Orthopaedic, Kabir Medical College, Peshawar, Pakistan; 4 Trauma Centre, Makhdoom Aali, Tehsil Dunyapur, Pakistan; 5 Department of Orthopaedics, Chandka Medical College, Larkana, Pakistan

**Keywords:** COVID-19, coronavirus, orthopaedics, safety measures, pandemic

## Abstract

**Introduction:**

The study aimed to target the current practices of the orthopaedic community in outpatient (OPD), emergency (ER) and surgical services (OT) during COVID-19.

**Material and method:**

This study surveyed 303 orthopaedic surgeons from all over Pakistan. The survey had 30 questions targeting the setup of outpatient, emergency and operation services in orthopaedic departments of different hospitals in Pakistan.

**Result:**

A total of 302 surgeons were included from 53 cities all over Pakistan. Between 35-48% of the respondents reported lack of availability of standard operating procedures in OPD, ER and in OT. Majority of the respondents noted that their OPD and surgical practice had been affected to some degree and 69% of the surgeons were only doing trauma surgery. This trend was higher in younger consultants of less than 45 years of age (p<0.001). Almost two-third of the surgeons, mostly senior (p=0.03) were using surgical masks as the only protective measure during various practices of OPD, ER and OT, while most of the setups were not assessing patients even for signs and symptoms of COVID. Almost 89% of the orthopaedic community is facing definite to mild stress during this pandemic and this has significantly affected the senior surgeons (p=0.01).

**Conclusion:**

Our study highlighted that COVID-19 has resulted in marked changes to the practices of the majority of Pakistani orthopaedic surgeons. Despite a sharp upsurge in the number of cases and mortality due to COVID-19, guidelines were still lacking at most of the settings and a substantial percentage of the orthopaedic community were not following adequate safety measures while attending to patients.

## Introduction

The recent pandemic of Coronavirus disease (COVID-19) has affected the lives of people worldwide. However, the greatest burden has been placed on the medical profession. It has posed a huge challenge to the medical units and teams working around the world^1,2^. While the front line health care workers are facing daunting tasks of handling COVID-19 patients, the other medical sub-specialties are struggling hard to manage and maintain their essential services and indispensable workflow^3^. The main aim is to minimise the effect of the COVID-19 pandemic on other patient care areas. As a part of the large health-care network, orthopaedic surgeons also have a critical role to play during this pandemic. They are at risk from a variety of sources in both inpatient and outpatient settings as they have close patient contact during the management of both non-operative and operative orthopaedic cases^4^. The findings of a recently published study endorsed this notion and reported that 78% infected healthcare workers were non-front liners^5^. Hence, proper measures and guidelines must be practiced to halt or at least minimise the spread of infection as much as possible while attending to the patients.

In the face of the current pandemic, the heath care facilities of a developing country like Pakistan are getting stretched thin. Medical resources like the number of doctors and nursing staff, particularly the specialists, the equipment and protective-gear all in combine give an alarmingly fragile picture^6^. Latest data shows a sharp upsurge of virus cases among health care workers and many deaths have been reported so far which is alarming^7^. Therefore, the careful prioritisation of trauma and orthopaedic surgical services must be planned keeping in view the clinical urgency, resource availability and protection of patient and healthcare workers^8^. The orthopaedic community in their own capacity is maintaining the work flow in different hospitals inclining to patients which require urgent or early orthopaedic care such as with musculoskeletal trauma and tumours^9^. In general the proper guidelines and safety measures must be taken into account to reduce the risk of infection in patients and medical personnel at different setups^10^, but as the case numbers are escalating further utmost care and caution is desirable.

In the current scenario, orthopaedic surgeons need to be aware of the hazards to patients and health care personnel in view of underdiagnosed cases. The understanding and protocols should be clear for pre-operative COVID-19 evaluation for all surgical cases^11^. Our local orthopaedic community in Pakistan varies widely with surgeons of extreme ages, expertise and resources, therefore, it is highly mandatory to assess and evaluate the current practices of orthopaedics in this COVID era for better understanding of existing health and safety measures. The current study has designed an anonymous online survey for orthopaedic surgeons of the country to gain valuable information regarding their setup during COVID-19 and to assess the practices they are following. This will help us to understand how the emergence of COVID-19 has impacted orthopaedic surgeons and the findings will lead us to guide policy makers to address the deficiencies and meet the current and future needs of our healthcare system.

## Material and Method

A survey consisting of 7 demographics, 18 other questions targeting outpatient (OPD), emergency (ER) and surgical practices, and 5 general questions Table I to understand the behaviour and state of mind of orthopaedic surgeons during this COVID duration was designed on Google forms survey. The survey had a total of 30 questions with 2 to 6 options each and was based on detailed discussion and agreement by the working group of 2 orthopaedic surgeons and 1 researcher. The survey link was sent via Emails, WhatsApp and Facebook to various orthopaedic surgeons in Pakistan using a non-probability snowball sampling technique. The survey was collected from 17th May to 5th June 2020 and reminders were sent on these platforms at various time points to participants to complete the survey. All categorical variables were presented as percentages. Data was entered in SPSS version 20 [IBM Corp] for statistical analysis. Chi square test was applied to see the statistical significance of age group on other categorical variables. Spearman rank correlation was used to evaluate relationships involving ordinal variables. Multiple logistic regression analysis was done to consider the simultaneous influence of several variables on the response. P-value < 0.05 was considered significant.

**Table I:** Table showing survey questions that were asked from the participants regarding their practice

#	Survey Questions regarding Practices in Outpatient, Emergency & Operation theatres
1	Has any SOPs for managing OPD been laid down by your department?
2	Is your work setting still following the Pre-COVID 19 routine of outpatient clinic?
3	If ‘No’ how it is reduced (check all that apply)
4	Is your own personal private OPD practice has been changed?
5	Which of the following are you using during patient care in OPD (check all that apply)?
6	Did you observe any change in the patient turn out in OPD?
7	Has your department started any telemedicine services during this pandemic?
8	Has any SOPs for managing emergency services been laid down by your department?
9	Emergency health care staff using which of the following during patient care (check all that apply)?
10	On arrival to emergency services patients are screened for COVID 19 signs and symptoms like temperature, cough etc
11	Has any SOPs for managing operation theatres been laid down by your department?
12	What type of surgeries you are performing?
13	Are you doing your private surgeries?
14	Which of the following are you using during surgery (check all that apply)?
15	Are you keeping your surgical team as minimal as possible?
16	Are you screening your patients for COVID-19 before surgeries?
17	Patients stay in hospital after surgical procedure has been minimized as much as possible
18	Are you trying to keep your surgical time short?
19	Have you attended/operated any COVID suspected/ positive patient?
20	How stressed out are you with the current scenario
21	Are you involved in teaching learning during this lockdown period?
22	Are you involved in any research during this lockdown?
23	Are you happy with your hospital response to COVID 19 pandemic?

## Results

**Fig. 1:**
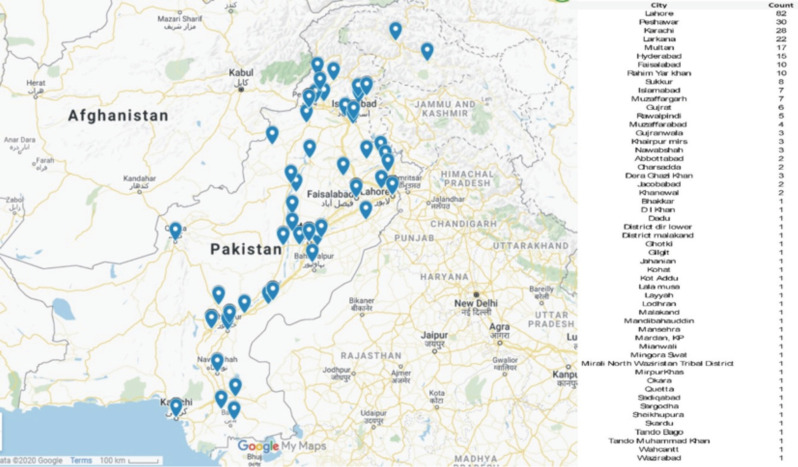
Representative map drawn on Google map showing distribution of survey responses received from various cities of Pakistan. https://www.google.com/maps/d/u/0/edit?mid=1D51I7_DtReSqUsvi4vlfTZXDmp5wcefK&usp=sharing (accessed on 06th June 2020).

The responses were collected from 53 cities all over Pakistan (Fig 1). In total, 303 individuals undertook the survey; one form that was completed by a foreign orthopaedic surgeon was excluded since the scope of the survey was to highlight the practices among Pakistani orthopaedic surgeons. Among the 302 respondents, 99% were males, almost 63% were between the ages of 25-34, 67% had completed their postgraduate training whereas the rest were residents and trainees. The demographic and work practice details are summarised in Table II.

**Table II:** Table showing respondents demographics and work setting details

Age					Completed PG training		Practice			Work setting			
25-34	35-44	45-54	55-64	> 65	Yes	No	Urban	Rural	Both	Outpatient Private / Specialty clinic	/ Private Hospital	Govt. Hospital	Multiple
62%	20%	9%	8%	1%	66%	34%	83%	13%	4%	22%	15%	42%	21%

**Fig. 2:**
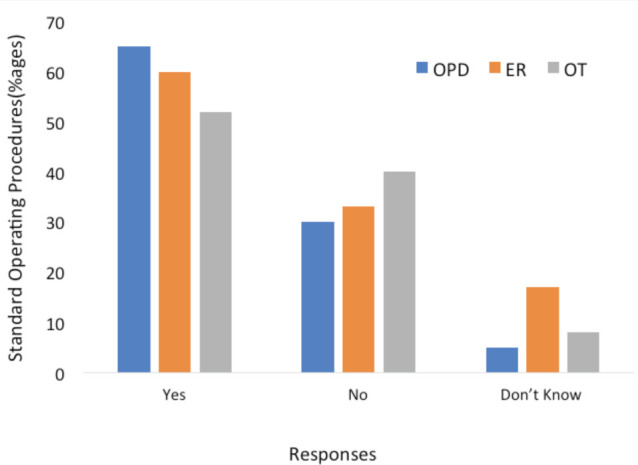
Bar graph based on the responses showing outpatient departments (OPD), emergency (ER) and operation theatres (OT) with and without standard operating procedures.

In response to the query regarding standard operating procedures (SOP) laid down by the department during the COVID-19 pandemic in OPD, ER and operation theatres (OT), almost 35% of the respondents mentioned lack of availability of SOP in their OPD, 40% stated this deficiency in ER and almost 48% in OT (Fig 2). Concerning the routine of the OPD, 75% of the doctors stated that they had changed their routine of OPD practice as compared to the pre-COVID era. Of these 30% had reduced the days, reduction in the staff had been done by 30% while 11% had reduced the timings. Private OPD practice had also been reported to be changed by almost 90% of the orthopaedic surgeons and almost 43% had stopped attending OPD while 40% were seeing only patients who expressed their urgent need.

**Fig. 3:**
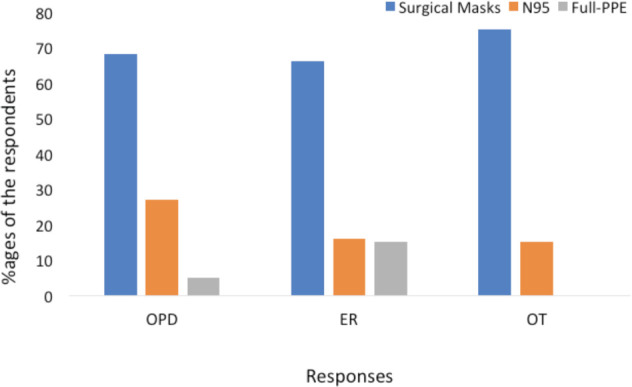
Bar graph showing percentage of respondents using various safety measures like only surgical masks, N95 respirator and full personal protective equipment (PPE) in outpatient departments (OPD), emergency (ER) and operation theatres (OT).

Regarding the safety practices in OPD, only 5% opted for full PPE suit while 68% were using surgical masks. Among them only 1% had combined it with a face shield and 1% were alternating it with an N95 respirator. However, only 27% were using N95 masks strictly in OPD, which drastically reduced to 16% in ER and 17% in OT. Majority of the respondents, that is 66% in ER and 75% in OT were using surgical masks only. In emergency services full PPE safety suit was only used by 15% of the individuals (Fig. 3). Interestingly the safety practices by the individuals were seen to be correlating in all the three areas of practice (p<0.001) Table III.

**Table III:** Spearman rank correlation coefficient for comparison between safety practices in three areas of practices

Safety Practices	OPD	ER	OT
OPD	-	0.38*	0.39*
ER	0.38*	-	0.21*

Abbreviations; OPD: Outpatient Department, ER: Emergency, OT: Operation Theatre, * P<0.001

Only 66% of the orthopaedic emergency services were screening patients for COVID-19 with signs and symptoms on arrival, while 25% of the respondents reported no screening at all and 9% did not have any clue regarding screening. Before surgery almost 58% of the orthopaedic surgeons were assessing the patients for COVID-19 mainly by signs and symptoms (51%); only a small percentage (7%) were taking swabs for PCR. The rest of the surgical community were not even assessing the patients for any signs and symptoms of COVID-19 before surgery. A total of 54% among them were not sure if they had operated on any COVID-19 patient or not, however, 10% confirmed to have operated COVID-19 patients.

In the current scenario of COVID-19 only 22% of the orthopaedic surgeons were doing both trauma and elective surgeries whereas 69% were doing only those trauma surgeries which could not be avoided and 9% were not doing any surgeries at all. Almost 64% had stopped doing surgery in private practice and 18% had minimised the number of surgeries performed. Nearly 70% of the orthopaedic surgeons were keeping their surgical time and team short and 78% had reduced the duration of patient’s stay in hospital after surgery.

In response to general questions; 59% of the respondents mentioned that their departments had taken the steps for the provision of telemedicine services during this pandemic. Regarding involvement in teaching and learning during this partial lockdown period, 79% confirmed their involvement mainly through webinars and zoom meetings and participation in research activity was only affirmed by 16% of individuals. In general 89% of the respondents indicated definite to mild stress and 63% showed dissatisfaction regarding the response of their hospitals to COVID-19.

There was a significant impact of age on the change in private practice (p<0.001), almost 60% of the orthopaedic consultants younger than 45 years had mentioned that they had stopped doing private surgeries compared to only 29% of the consultants above 45 years. Multiple logistic regression showed the strongest predictors of age (p=0.001) and completion of postgraduate training (p<0.001) in private practice surgery.

The age also has a significant effect on the use of protection gear during OPD services (p=0.03), the younger age group of orthopaedic surgeons were more careful and used better protection compared to the group above 45 years of age. The orthopaedic surgeons working in rural setting were found to be more careful in practicing safety measures while working in OT (p=0.003) using N95 mostly. Multiple logistic regression showed the strongest predictors of practice setting in rural area (p=0.01) in the use of protection measures. With increasing age level of stress was significantly higher (p=0.01), the orthopaedic surgeons above 45 years of age showed higher level of stress compared to the group below 45 years who were mostly mildly stressed. Multiple logistic regression analysis showed that the strongest predictor was of age (p=0.01).

## Discussion

This study represents the first attempt to identify the OPD, ER and surgical practices among orthopaedic surgeons during the COVID-19 pandemic in Pakistan. Based on the responses it was noted that a huge number of hospitals and health care settings still lack SOP in their outpatient departments, emergency areas and especially in operating rooms. With this global pandemic, healthcare systems around the world are preparing themselves to take care of surgeries at more than maximum capacity for a number of months, therefore, proper guidelines and safety training in surgical services are crucial at this stage^10,12-14^. In these unique circumstances it is mandatory that hospitals and surgical units make comprehensive SOP which can be adapted by surgeons^2^.

Pakistan Orthopaedic Association (POA) has issued the “Standard Operating Procedure for COVID-19 Coronavirus Patients”^15^ which should be strictly followed in hospitals and every orthopaedic surgeons should be made aware of, however, these guidelines need improvement and have failed to set the protocols to be followed in operating theatres. Many international surgical and orthopaedic departments and societies have already provided a frame work for the elective and urgent surgeries and careful planning has been introduced to minimise staff shortages related to uncontrolled viral spread^13,16^. Since Pakistan is far behind in the number of people tested for COVID-19 there is an estimate that there is huge number of asymptomatic patients in the local settings. A recent study done at an orthopaedic specialty hospital in New York City reported that 59% COVID positive patients were asymptomatic among those screened prior to their planned orthopaedic surgical procedure^17^. This shows the extreme likelihood of asymptomatic patients and the potential risk of transmission of the disease. Therefore, vigilant control measures are warranted at every step considering every patient as a possible source of infection.

Regarding the safety protocols, the responses of the orthopaedic surgeons were quite alarming in all the settings. It was revealed that most of the orthopaedics are using only surgical masks while attending patients, evidence suggests that surgical masks are not a reliable protection against COVID-19^8^. The current survey further revealed that senior consultants above the age of 45 years have a more casual attitude towards self-safety measures and this raises the additional concerns with the possibility for them becoming infected and requiring self-isolation. This could potentially lead to a dangerous shortage of senior expertise within surgical teams and may increase the risk of cross infection to patients and others.

Healthcare professionals are quite vulnerable to the exposure of aerosols in the theatres, therefore, it is advisable for them to wear N95 masks and proper gear even during surgery^4,10^. It was quite interesting to find that in the rural health settings orthopaedic surgeons are more careful while operating as compared to urban settings. In the current scenario every patient should be treated as a potential COVID-19 positive case and it is logical that a surgical balaclava, gown and double gloves must be used as standard practice to minimise potential skin contact with the virus^4^. The guidelines published recently for orthopaedic departments on the basis of experience from Wuhan, People’s Republic of China highly recommend that in OPD “orthopaedic surgeons should select appropriate personal protective equipment (PPE) based on Level-II protective standards, including a hair net, gloves, an isolation suit, a medical respirator (filtering face piece [FFP]/N, R, or P), eye protection (goggles and/or visor), and shoe covers”^18^. Recently a case report of a 64-year-old male patient who was tested positive for SARS-COV-2 and underwent explantation of a shoulder prosthesis due to a periprosthetic infection suggests that it is possible to prevent spread of infection among health care workers if strict control measures are taken. In this case all 66 health care workers remained safe and COVID-19 was not detected in any of them despite high-risk exposure during intubation, surgical treatment and general care^19^.

It was indicated through our survey that in most of the settings the screening of patient is usually through assessment of signs and symptoms leaving the gap of asymptomatic transmission and putting orthopaedic surgeons at high risk of infection. The recommendations of POA regarding the dress code and 3 ply surgical masks when not in COVID wards^15^ is quite perilous in these circumstances.

A large number in the orthopaedic community displayed higher level of stress and this was significantly higher in senior consultants above the age of 45 years. The stress may be due to loss of practice, financial burden and future uncertainty as most of the surgeons have either stopped their surgical practice and/or reduced it and were not happy with the steps taken by their administration in response to COVID. A disrupted life work balance with loss of financial support, emotional stress, fear of falling sick and health risks for the family could be factors for the mental stress in the orthopaedic fraternity^20,21^.

## Conclusion

Our study strongly suggests that appropriate use of protective gear and infection control protocols are essential among the orthopaedic fraternity especially at this stage when Pakistan is facing a major outbreak of virus cases these days. The concerned societies and hospitals should take serious steps to outline and define the standard protocols at every step to minimise the transmission and cross infection.
